# Making the invisible visible: a systematic review of sexual minority women’s health in Southern Africa

**DOI:** 10.1186/s12889-016-2980-6

**Published:** 2016-04-11

**Authors:** Alexandra Muller, Tonda L. Hughes

**Affiliations:** Gender Health and Justice Research Unit, University of Cape Town, Health Sciences Faculty, Falmouth Building, Room 1.01.5, Anzio Road, Observatory, Cape Town, 7925 South Africa; Global Health University of Illinois at Chicago College of Nursing (M/C 802) Room 1160 Chicago, ᅟ, IL 60612-7350 USA

**Keywords:** Sexual minority health, Women’s health, Southern Africa, Systematic review

## Abstract

**Background:**

Over the past two decades research on sexual and gender minority (lesbian, gay, bisexual and transgender; LGBT) health has highlighted substantial health disparities based on sexual orientation and gender identity in many parts of the world. We systematically reviewed the literature on sexual minority women’s (SMW) health in Southern Africa, with the objective of identifying existing evidence and pointing out knowledge gaps around the health of this vulnerable group in this region.

**Methods:**

A systematic review of publications in English, French, Portuguese or German, indexed in PubMed or MEDLINE between the years 2000 and 2015, following PRISMA guidelines. Additional studies were identified by searching bibliographies of identified studies. Search terms included (Lesbian OR bisexual OR “women who have sex with women”), (HIV OR depression OR “substance use” OR “substance abuse” OR “mental health” OR suicide OR anxiety OR cancer), and geographical specification. All empirical studies that used quantitative or qualitative methods, which contributed to evidence for SMW’s health in one, a few or all of the countries, were included. Theoretical and review articles were excluded. Data were extracted independently by 2 researchers using predefined data fields, which included a risk of bias/quality assessment.

**Results:**

Of 315 hits, 9 articles were selected for review and a further 6 were identified through bibliography searches. Most studies were conducted with small sample sizes in South Africa and focused on sexual health. SMW included in the studies were racially and socio-economically heterogeneous. Studies focused predominately on young populations, and highlighted substance use and violence as key health issues for SMW in Southern Africa.

**Conclusions:**

Although there are large gaps in the literature, the review highlighted substantial sexual-orientation-related health disparities among women in Southern Africa. The findings have important implications for public health policy and research, highlighting the lack of population-level evidence on the one hand, and the impact of criminalizing laws around homosexuality on the other hand.

**Electronic supplementary material:**

The online version of this article (doi:10.1186/s12889-016-2980-6) contains supplementary material, which is available to authorized users.

## Background

Over the past two decades research on lesbian, gay, bisexual and transgender (LGBT) health has highlighted substantial health disparities based on sexual orientation and gender identity in many parts of the world. Although interest in sexual minority (SM) health has disproportionately focused primarily on sexually transmitted infections—in particular HIV/AIDS—there is growing awareness of the broad ranging negative health consequences of stigma, marginalization and discrimination among SM people [1–5]. For example, in a recent landmark American report on SM health [3], the Institute of Medicine (a US non-profit, non-governmental organization) pointed out that SM people are at increased risk of harassment, victimization, depression and suicide and have higher rates of smoking and alcohol use than their heterosexual counterparts. The report further indicates that lesbian and bisexual women may also be at higher risk for obesity, cardiovascular disease and breast cancer. These findings underscore the link between stigma, marginalization, discrimination and health outcomes [6, 7], and corroborate that sexual orientation is an important social determinant of health [4].

Most of what is known about SM health is based on research conducted in high resource countries—especially in the US. Further, existing research is heavily skewed toward SM men—and is disproportionately focused on HIV and other sexually transmitted infections. For example, in a review of research grants funded by the US National Institutes of Health (NIH) between 1989 and 2011, Coulter and colleagues [8] found that aside from studies of HIV/AIDS, only 0.1 % of all funded studies addressed SM health. Of these, most focused on gay and bisexual men; only 13.5 % of studies on SM health focused on lesbian or bisexual women. Similarly, in a review of English language articles indexed by MEDLINE between 1980 and 2000, Boehmer [9] found the same low proportion of research (0.1 %) focusing on SM health issues, and of this proportion, only 37 % included information about lesbian or bisexual women. Many of these articles addressed lesbians, bisexual women, and transgender persons as a group, despite known differences in their health risks and outcomes. While lesbian and bisexual women share with transgender women many of the vulnerabilities due to social exclusion and stigmatization, the fundamental difference between sexual orientation (lesbian or bisexual) and gender identity (transgender) also lead to significantly different health needs, for example access to gender affirming health care for transgender people [10].

A report by the Executive Board Secretariat of the World Health Organization points out that one of the main challenges to improving the health and well-being of SM people is the “institutional prejudice, social stress, social exclusion (even within families) and anti-homosexual hatred and violence…” that they face. The report goes on to say that in order to “achieve a better understanding of the health needs of LGBT people, more data are needed on the demographics of these populations, particularly in low-income and middle-income countries…” The report emphasizes that as a first step there needs to be a rigorous and systematic review of the literature [5].

Over the past few years, accounts of homophobic sexual assault of SMW (often problematically labeled ‘corrective rape’) have increasingly been reported from civil society organizations in South and Southern Africa [11–13]. While South Africa has one of the highest prevalence levels of sexual violence worldwide [14], assaults against SMW are marked by the homophobic motivation of the perpetrator(s), who claim that rape will ‘cure’ lesbian and gender non-conforming women (or women who are perceived as SMW) from their homosexuality [13]. Women of color who live in resource-poor peri-urban areas are particularly targeted [11–13].

Such homophobic sexual violence is symptomatic of the social exclusion and homophobia in which SMW live in the region. With the exception of South Africa, Southern African countries tend to have very restrictive laws that either criminalize homosexuality, or at least do not offer any special protection to sexual minority groups [15]. The vast majority of people in Southern African have grown up in patriarchal heterosexist (biased in favor of opposite-sex sexuality and relationships) societies that have little recognition of SMs in general, and of SMW in particular. As elsewhere in the world, societies are characterized by hegemonic forms of masculinity that regard heterosexuality and homophobia as the bedrock of masculinity, which, in turn, may play a role in perpetuating the lack of recognition of (and violence against) sexual and gender minorities [16]. This can be seen in the media, religion, legal discourses, education and health care. Sexual orientation is, for example, very seldom discussed even when educating about the risks of HIV transmission, effectively rendering SMW invisible [17]. This silencing of sexual and gender minorities is powerful, as it implies taboos and undesirability, and perpetuates prejudice towards this population [18].

The social context in which sexual and gender minorities in Southern Africa live varies based on race, gender, class and socio-economic status and geographical location. Sexual and gender minorities in Southern Africa, as elsewhere, are not a homogeneous group. For example, in South Africa the effects of colonialism and apartheid are significant, and the white population is significantly better educated and more economically stable than South Africans of color [19]. Although there have been some gains, gender inequality is still apparent due to the patriarchal nature of South African society, and researchers have highlighted that SMW have unique challenges due to their double marginalization as women and sexual minorities [20]. Black lesbian women are therefore especially vulnerable to marginalization due to membership in multiple oppressed groups [21]. Furthermore, the health systems in Southern African countries, unlike in Europe and the US, are built on a primary health care model rather than emphasizing tertiary care. Despite this, the majority of health systems are severely under resourced [22].

In summary, the context in which Southern African SMW live is unique in regard to the socio-political climate, legislative framework, impact of apartheid and colonialism and the influences of race, gender, class, and other markers of oppression [23]. Although too little research has been done to determine how these factors influence the health of sexual minorities, it seems reasonable that sexual-orientation-related health disparities exist in Southern Africa as in other countries and contexts. Understanding health disparities is crucial to the development of effective and protective health policy, and to the design of relevant, appropriate public health interventions aimed at decreasing disparities and improving the health and well-being of SMW. In this paper we report the results of our effort to systematically review the literature related to the health of SMW in Southern Africa, with the objective of identifying existing evidence around health issues and health risks for this vulnerable group, including perceived health concerns, health disparities, prevalence of health conditions and patterns of health-seeking behavior.

## Methods

Southern African countries were defined to include South Africa, Namibia, Botswana, Zambia, Zimbabwe, Lesotho, Swaziland and Mozambique. These countries share a cultural and social history, have close socio-economic relationships with each other, and experience similar challenges in health and health service provision. We followed the PICOS approach [24] to define the review question as: What evidence exists for health issues and health risks for SMW in Southern Africa? Prior to the search, a decision-making matrix was created to define search terms, inclusion and exclusion criteria, and the overall search process [25]. All empirical studies using quantitative or qualitative methods that contributed to evidence for SMW’s health in one, a few or all of the countries, were included. Theoretical and review articles were excluded. SMW were defined to include lesbian women, bisexual women, and women who have sex with women (WSW). Although transgender women share with SMW many of the vulnerabilities, they were excluded from this review to avoid a conflation of health outcomes and needs specific to gender identity with those related to sexual orientation. All studies with LGBT populations were included if they provided information about the number of SMW in their samples. Articles in English, French, Portuguese or German were included for the years 2000 – 2015.

We used the following search strategy based on an initial review of keywords in international literature on SMW health. We used search terms for previously identified key health issues for SMW [3, 26]:

- (Lesbian OR bisexual OR “women who have sex with women”) AND
- (HIV OR depression OR “substance use” OR “substance abuse” OR “mental health” OR suicide OR anxiety OR cancer) AND
- (“South Africa” OR “Southern Africa” OR Africa OR Namibia OR Zambia OR Zimbabwe OR Lesotho OR Botswana OR Swaziland OR Mozambique).

We searched PubMed and MEDLINE databases with all possible combinations of search terms for keywords and indexing terms. Searches were conducted on 5^th^ and 7^th^ February 2015. The title and abstract of all articles identified in this initial search were reviewed by one of the authors. Articles that matched the inclusion criteria were reviewed in full. In order to identify more potential articles, the references of all included articles were reviewed for further articles that matched the inclusion criteria. Once identified, the screening process described above was employed to determine whether they should be included. Furthermore, we contacted the authors of existing publications to ask about any further publications or reports.

Data were extracted from the identified publications using a data extraction form (see Additional file 1), covering study identification information (author, date, country of origin), study characteristics (study design, data collection methods), sample characteristics and demographic information (number of enrolled participants, age, gender, sexual orientation, and other characteristics as reported), results (prevalence of sexual health/mental health/violence/other health issues, and factors associated with each), and an assessment of risk of bias/quality. For quantitative studies, attrition and the potential of reporting bias were examined [27]. For qualitative studies, quality was determined based on theoretical basis, sampling strategy, scope of data collection, description of data collected, and concern with transferability or typicality [28, 29]. Risk of bias and quality were classified as low, unclear or high [28]. Data were extracted by one of the authors (AM) and a research assistant. The PRISMA checklist for this systematic review is available in Additional file 2.

## Results

**Fig. 1:**
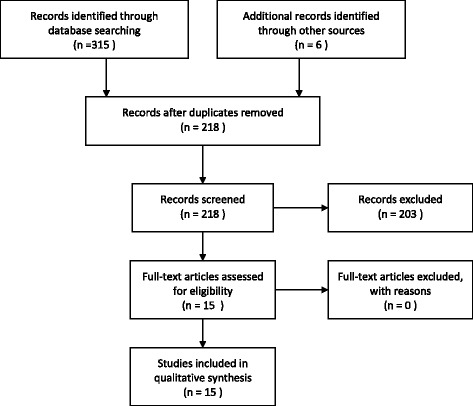
Search strategy and results. Search strategy according to guidelines from the PRISMA statement [24]

The initial search yielded 315 hits [see Additional file 3 for the full PubMed search]. Figure 1 provides an overview of the search and selection process. After screening for duplicates, 208 articles were excluded from the review because they did not provide data on SMW (*n* = 103), did not provide data from Southern Africa (*n* = 62), or because they did not present data from empirical qualitative or quantitative studies (*n* = 38). Nine articles were selected for this review. A further 6 articles were identified through reference lists of selected articles. An additional table provides an overview of the 15 articles and key findings [see Additional file 1]. Eight studies used quantitative methodology [30–37], one triangulated the results of a mixed methods study [38], and six employed qualitative methods [39–44]. All quantitative studies were classified as having an unclear risk of bias [30–37]; of the qualitative studies two were classified as high quality [39, 42] and the rest as unclear [40–44].

Most articles present data from South Africa; we found no studies from Swaziland, Zambia or Mozambique. Articles published before 2013 (*n* = 6) focused mostly on mental health and well-being, while those published after 2013 (*n* = 9) focused on sexual health and risk of HIV. Sample sizes ranged from 11 to 591 participants. All quantitative studies relied on participant self-report. None of the studies used longitudinal research designs. While 7 articles focused exclusively on women (WSW, lesbian and bisexual women), the other 8 either presented data from studies with lesbian, gay, bisexual and transgender participants (*n* = 6) or heterosexual and sexual minority participants (*n* = 2).

### Characteristics and Help-Seeking Behaviors of SMW in Southern Africa

The mean or median age of SMW in most of the studies was between 23 and 27 years [30, 31, 35, 37–39, 41]. Four studies focused specifically on youth below the age of 24 [33, 36, 43, 44]. SMW participants were heterogeneous in racial and socio-economic composition. Ten studies reported the race of participants, which was representative of their respective national context [30, 31, 34–39, 43, 44]. Between 43 % [38] and 47 % [30] of SMW in the 15 studies reviewed reported consensual sex with both women and men.

Results indicate that SMW were reluctant to use health care services [31, 37, 39], including preventive services: for example, only 12 % in Poteat et al.’s [38] sample had had a pap smear in the past 2 years. As a result, a number of studies found that SMW did not have adequate sexual health knowledge [39, 42] or did not know where to search for information regarding their health [40]. In addition, SMW were also reluctant to disclose their sexual orientation or information about their sexual activity to health care providers [32, 38, 39]. Although 78 % of SMW in Sandfort et al.’s [30] sample had tested for HIV, of those who had not, 39 % thought they were not at risk. Furthermore, young SMW often hid their sexual orientation in institutional spaces like schools and health facilities [44] and as a whole SMW often did not report experiences of hate victimisation to the police [34].

### Health issues among SMW

Most of the studies reviewed focused on sexually transmitted infections, particularly HIV. Self-reported HIV prevalence ranged between 8 and 13.8 % [30, 33, 38]. The eight studies that reported experiences of sexual violence found that 8 to 31 % of women reported lifetime experiences of non-consensual sex [30–32, 34, 36, 42]. Sandfort and colleagues [31] elaborate that of 31 % of SMW in their study who reported forced sex, 14.9 % reported forced sex by men only; 6.6 % reported forced sex by women only; and 9.6 % had forced sex experiences with both men and women. Compared to white SMW, sexual violence levels were notably higher among black SMW [34], who were more likely to experience violence in public spaces, and less likely to report it to the police [37]. Qualitative accounts of sexual violence indicate that perpetrators often made it clear that such violence was justified as a ‘corrective’ measure, and that it was a way of enforcing heteronormative, patriarchal structures. As one study participant noted: “They [male students] said that it would make me ‘a real lady’. They stressed that corrective rape was the best way to change my satanic behaviour” [41].

Eight of the studies reviewed included a focus on mental health. Kowen and Davis [43] and Polders et al. [35] show the impact of heteronormativity and social exclusion on SMW’s mental health, particularly as it relates to psychological distress and elevated rates of depression. Experiences of hate speech [34], sexual violence [31] and religion-based stigma and discrimination [41] were associated with mental distress and suicidal ideation among SMW. Compared with their age-matched heterosexual peers, SMW youth showed higher levels of traumatic stress and substance use [36]. Substance use prevalence ranged from 50 % for recreational drugs [30] to 64 % for daily alcohol use [32].

### Social exclusion and social determinants of health

A number of studies report on the context of social exclusion, including exclusion from socio-economic opportunities, and highlight the invisibility of SMW in health service provision and preventive health care [30, 39, 44]. Butler and Astbury [44] explore how young participants ‘covered up’ and denied their sexuality in order to conform to society’s norms. A study conducted on a rural South African university campus [41] found that organised religion played a crucial role in justifying exclusion and discrimination of SM students. All women in Smith’s [39] qualitative study stressed the lack of sexual health information, and lack of health care providers’ knowledge, which they perceived as tied to the general invisibility of SMW women.

## Discussion

This review highlights substantial sexual-orientation-related disparities and many gaps in information about SMW’s health in Southern Africa. Although research focusing on SMW’s health in this region of the world is extremely limited, the 15 studies that we were able to locate provide a beginning understanding of Southern African SMW’s health status and health risk factors, and highlight important public health implications.

As in studies in other parts of the world, mental health issues figured prominently in the studies reviewed. Elevated rates of depression, substance use and other mental health issues (e.g., suicidal ideation) reported in the studies reviewed here must not be assumed to be inherent in being a SM person [45]. Rather these and other health disparities are the likely result of stigma, marginalization and invisibility; stress associated with hiding one’s sexuality; and/or the impact of enduring verbal, emotional, physical and sexual abuse from intolerant peers, family and community members [7].

Although little is known about the actual prevalence of sexual violence experienced by SMW, findings from studies reviewed here support numerous non-academic sources which suggest that SMW in Southern Africa are particularly vulnerable to sexual assault [46, 47]. Although civil society has begun to document individual accounts of sexual violence against SMW, academic research is urgently need to provide data about prevalence, risk factors and outcomes (e.g., pregnancy, sexually transmitted infection, post-traumatic stress) of sexual assault and to understand how to provide competent and comprehensive support for SMW survivors in the health and criminal justice systems. Such information is also essential to the development of prevention strategies aimed at reducing violence against SMW.

Findings from the review suggest that Southern African SMW, like their counterparts in other parts of the world, are reluctant to engage with the healthcare system—and consequently are less likely to receive important preventive health screenings [44, 45] and other needed health services. Studies in the US show that sexual and gender minorities encounter a number of barriers to accessing care and that these barriers generally fit into one of four main themes: (1) reluctance by some SMs to disclose sexual or gender identity when receiving care, (2) insufficient numbers of healthcare providers who are competent in dealing with SM people and their unique health concerns, (3) structural barriers that limit access to health insurance and healthcare decision-making rights of SM people and their partners, and (4) a lack of culturally appropriate prevention services [48]. Given the stigma and marginalization faced by SM people in most parts of Southern Africa these barriers likely exert an even stronger impact, and are compounded in countries where same-sex sexuality is a criminal offense [15]. Such laws negatively impact access to health and criminal justice services, and increase discrimination and violence against SMW [49].

SMW have multiple and good reasons for not disclosing their sexual or gender identity to providers including concerns about confidentiality and fears of homophobic reactions or being further stigmatized—often based on negative past negative experiences with providers [48]. As Mayer and colleagues note, to the extent that these concerns cause SM clients to delay help-seeking or to withhold information that may be important to treatment, effective health care can be compromised [48].

Whereas a scant minority of studies of SMW’s health in the US, Canada and Australia focus on sexually transmitted infections, and even fewer focus on risks of HIV, the majority of the studies reviewed here focused on risky sexual behavior—especially in the context of HIV. However, as previous authors have argued, SMW are marginalized even within HIV research. Logie and Gibson [50] point out that SMW are often erased from discourse around violence and HIV in North America and that the way they do (or do not) appear in HIV discourse says a great deal about views of women, sexuality and the global HIV/AIDS pandemic. Richardson [51] asserts that the invisibility of SMW in research related to HIV is often due to the (erroneous) belief that women who have sex with women are not at risk for sexually transmitted infections, and leads to socially constructed ‘immunity’ based on the assumption of a close relation between sexual behavior and sexual identity. It is becoming increasingly clear that substantial proportions of lesbian and other sexual minority women engage in sex (both consensual and forced) with male partners. Based on findings from the studies reviewed here nearly one-half or more of SMW reported having consensual sex with men. Having concurrent female and male partners has been found to be significantly associated with HIV infection [28]. Nevertheless, the two studies that reported rates of HIV among SMW were both lower than the 14.4 % prevalence in the general female population in South Africa [52], the country with the highest HIV prevalence in the region. Given that all of the studies reviewed here relied on participant self-report to collect data—and given SMW’s limited access and reluctance to seek care [34, 39, 40]—these findings likely underestimate HIV and other sexually transmitted infections.

Matebeni et al. [42] and Sandfort et al. [31] highlight the impact of HIV infection on SMW’s sense of belonging and physical and mental health. Matebeni et al.’s [42] suggestion that SMW living with HIV are often excluded from SM communities poses important questions around access to resources and support for SMW living with HIV, and further emphasize the need for research aimed at understanding SMW’s specific vulnerabilities and access to services and health needs, including psychosocial needs around HIV infection and antiretroviral medication adherence. Unfortunately, as Smith [39] notes, limited knowledge among health care providers precludes a nuanced understanding of sexual behavior and sexual identity, and therefore sexual risk behaviors among SMW are seldom addressed in sexual health education and HIV counseling.

Existing information about Southern African SMW’s health comes from either a public health or social science perspective. Few of the studies reviewed mentioned issues of intersectionality. Dworkin [53] stresses the importance of analyzing intersecting identities when researching HIV risk for lesbian and bisexual women by highlighting that sexuality is not the only factor that places lesbian and bisexual women at risk for HIV: it is well established, for example, that poor, marginalized women of color living in inner city areas in the US have been disproportionally affected by HIV [54]. Similarly, SM women’s vulnerability to sexual violence and HIV in South Africa appears to be heavily shaped by race and class [13]. Such vulnerability is not captured in analyses that focus exclusively on sexual orientation and/or gender identity. More interdisciplinary work is needed that takes into account SMW’s intersecting vulnerabilities and complex sexual identities and behaviors often not captured by epidemiologic surveys. At the same time, social science research that highlights such dynamics needs to be conducted at population-level to produce the quantitative evidence needed for the design of public health interventions and health advocacy.

Our review has several limitations. First, the two databases through which the search was conducted are medicine/public health focused, and might have missed studies from other disciplines such as psychology. Further, the lack of search terms for specific health issues might have limited the identification of studies that report on, but are not directly focused on such issues. Data screening and abstraction was performed was not double-checked by a second researcher, which increased the risk for bias in the process of choosing records for inclusion. Lastly, while studies in four languages (English, French, Portuguese and German) were included into our review, the English search terms might have missed studies indexed in languages other than English.

Second, the design and methodology of the studies identified for the review varied. Whilst all of the studies reviewed met the inclusion criteria, there were significant discrepancies in the level of detail in reporting study methodologies. Specifically, all quantitative studies relied on participant self-report, and no randomized control trials or longitudinal studies were found. Further, sample sizes varied considerably across the quantitative studies. In the review table, this information is specified for each reviewed study (Additional file 1). Risk of bias (for quantitative studies) and quality (for qualitative studies) could often not be determined because the publications lacked information on one or more aspects of study design and attrition. These individual study limitations significantly affect the assessment of the quality of the data reviewed, and bearing these limitations in mind, no meta-analysis was performed on the extracted data. The limitations at study and review level speak further to the limited research interest and capacity dedicated to examining SMW’s health that the review evidenced.

## Conclusions

This review identifies key areas of concern for future public health research for SMW health in Southern Africa. Given that homosexuality is illegal in most Southern African countries, legislative and policy changes are key to improving SMW’s health. Further, the knowledge, attitudes and skills of healthcare providers need to be improved to ensure culturally appropriate, quality care to SMW [55]. As health professions education currently does not provide such training [56], capacity for locally-adapted curricula [57] urgently needs to be built.

As Parker [58] points out, it is not only possible but also necessary for researchers to interrogate and challenge the existing heteronormative cycle of discursive and social exclusion of sexual minorities. However, as important as it is, research alone cannot address the inequities, violence, and health disparities faced by SMW—nor can it improve health care systems’ responses to SM people. Promoting the health of SMW in Southern Africa will require new judgments of social worth and a new political will. Even though much has been achieved in addressing issues important to SMW in many parts of the world, researchers, advocates and healthcare providers have much work to do to address the massive sexual-orientation-related health disparities and invisibilities in Southern Africa. As the South African Academy of Science concluded in a recent review of diversity in human sexuality: “To promote human welfare, we must advance two important goals: well-being and social justice. Recognizing the harm of bullying and other exclusionary behaviors and the damage caused by physical violence and fear in LGBTI [lesbian, bisexual, gay, transgender and intersex] communities, scientists in Africa should engage more actively in research to reduce stigma, and work further to promote access to health care and educational materials for LGBTI communities” [59].

### Ethics approval and consent to participate

Not applicable.

### Consent for publication

Not applicable.

### Availability of data and materials

The dataset supporting the conclusions of this article is included within the article (and its additional files 1 and 3).

## Additional files

Additional file 1: Overview of reviewed journal articles and key findings. Table of reviewed journal articles, with information on country, demographics, and key findings. (PDF 321 kb) Additional file 2: PRISMA checklist. Checklist for PRISMA criteria for systematic reviews. (PDF 191 kb) Additional file 3: Full search (PubMed). Details of full search strategy and results. (PDF 201 kb)

## Abbreviations

LGBT: Lesbian, Gay, Bisexual and Transgender
LGBTI: Lesbian, Gay, Bisexual, Transgender and Intersex
SMW: sexual minority women
WSW: women who have sex with women
