# Supplementary material for: Making the invisible visible: a systematic review of sexual minority women’s health in Southern Africa
Source: BMC Public Health. 2016 Apr 11;16:307. doi: 10.1186/s12889-016-2980-6 (PMC4827176; doi:10.1186/s12889-016-2980-6)
Supplement: Additional file 1: — Overview of reviewed journal articles and key findings. Table of reviewed journal articles, with information on country, demographics, and key findings. (PDF 321 kb) [file 12889_2016_2980_MOESM1_ESM.pdf]

Additional file 1

Author	Sample	Data collection method	Demographic information	Sexual health	Mental health	Violence	Other findings	Risk of bias/ Quality
Butler & Astbury, 2008 [44]	18 self-identified gay or lesbian youth in South Africa	QUAL Semi-structured interviews	Age 16 – 21 years<br><br>5 black, 2 'coloured', 2 indian, 4 white<br><br>4 lesbian women		Participants 'covered up' and denied their sexuality, withdrew physically and socially, acted as if they were straight, remained silent regarding their sexual orientation			Quality unclear
Ehlers et al. 2001 [32]	47 gay men, lesbian women & bisexual men and women in Botswana	QUANT Self-administered questionnaire	47% between 20 and 29 years<br><br>No info on racial composition<br><br>79% employed, 10% students<br><br>5 lesbian & bisexual women		30% reported suicidal thoughts at some stage, 30% on-going suicidal thoughts<br><br>64% reported drinking more than 2 drinks a day	11% had been sexually assaulted (no info on gender)	Only 15% of GLBs did disclose their sexual orientation to health care provider	Risk of bias unclear
Kowen & Davis, 2006 [43]	11 lesbian youth in South Africa (Cape Town)	QUAL In-depth interviews	7 Xhosa (black) youth aged 16-24 years<br><br>4 English (white) youth aged 16-19 years		Lesbian youth regularly face social isolation and rejection but are highly resilient			Quality unclear
Matebeni et al. 2013 [42]	24 self-identifying lesbians living with HIV in South Africa, Namibia, Zimbabwe	QUAL In-depth interviews	Age between 22 and 48 years<br><br>No info on racial composition<br><br>7 participants in full-time employment			8 out of 24 women (1/3) had been raped	Lack of knowledge about HIV transmission, risk and protection	Quality high
Mavhandu-Mudzusi & Sandy, 2015 [41]	20 LGBT students from rural SA university (5 lesbian and 3 bisexual women)	QUAL Semi-structured interviews using Interpretative Phenomenologi cal Analysis (IPA)	Age 19 – 25 years<br><br>No information about race or SES		Students stressed that homophobic labelling, stigma and discrimination led to suicidal ideation and suicide attempts	Lesbian students recounted threats of sexual violence to 'correct' their sexuality<br><br>Students shared threats of murder by heterosexual students	Religion-based prejudice led to stigma and discrimination of LGBT students by university staff (admin and lecturers) students and health care providers	Quality unclear

Additional file 1

Author	Sample	Data collection method	Demographic information	Sexual health	Mental health	Violence	Other findings	
Miller et al. 2013 [33]	830 South African youth (14 – 19 years old), 29 identify as lesbian, gay or bisexual (LGB)	QUANT Cross-sectional survey that focused on socio-behavioural characteristics, HIV status and SRH	Median age 17 years<br><br>No further demographic information	Self-reported HIV prevalence 13.8% of LGB identified youth (n=29), not disaggregated by gender				Risk of bias unclear
Muranda et al. 2014 [40]	54 WSW from Namibia, Kenya, Zimbabwe, Nigeria, Ghana, South Africa	QUAL Feminist virtual ethnography or 'internetography'	No demographic information	35% felt they were at risk for HIV/STIs<br><br>'Some' WSW engage in high-risk sexual behaviour which places them at an increased risk for HIV transmission			More than 50% had not visited a clinic or consulted with a healthcare worker on sexual health.<br><br>46% did not know where to get information regarding safe sex/ barrier methods for sex with women	Quality unclear
Nel & Judge 2008 [34]	487 lesbian and gay (86%) or bisexual (14%) respondents in South Africa	QUANT Self-administered questionnaire	46% between 15 and 24 years; 48% between 25 and 40 years<br><br>52% identified as black, 41% as white		37% of respondents feared violence on account of their sexual orientation, which lead to increased suicide ideation	37% had experienced verbal abuse,<br>16% physical abuse and 8% sexual abuse	Fear of discrimination can lead to the avoidance or delaying of accessing healthcare services and/or criminal justice services	Risk of bias unclear
Polders et al. 2008 [35]	385 gay and lesbian people (51.1% lesbian women) in Gauteng, South Africa	QUANT Self-administered questionnaire	Mean age 25 years (range 16 – 40)<br><br>79% identified as black, 21% as white<br><br>15% unemployed		Self-esteem and hate speech had strongest influence on vulnerability to depression			Risk of bias unclear

Additional file 1

Author	Sample	Data collection method	Demographic information	Sexual health	Mental health	Violence	Other findings	
Poteat et al. 2014 [38]	271 WSW in Lesotho	QUANT & QUAL Structured questionnaire (n=250), Focus group discussions (n=21)	Mean age 24 years (range 18-52)<br><br>98% ethnic Basotho<br><br>One third currently employed, 64% had received tertiary or vocational school education	Self-reported HIV prevalence 8%<br><br>76% had regular female partner, 43% had regular male partner<br><br>12% had had pap smear in past 2 years			Self-reported HIV infection significantly associated with having concurrent female and male partners<br><br>Only 25% of participating women had disclosed their same-sex behaviour to a health care worker	Risk of bias/ quality unclear
Sandfort et al. 2015 [31]	591 biologically female WSW in South Africa, Namibia, Botswana, Zimbabwe	QUANT Self-administered questionnaire, community-based	Mean age 26 years (range 18-65)<br><br>79% identified as black, 12% 'coloured', 6% white, 3% indian		Forced sex increased drug use, mental distress and lower sense of belonging	31% of participants reported that they experienced forced sex at least once		Risk of bias unclear
Sandfort et al. 2013 [30]	591 biologically female WSW in South Africa, Namibia, Botswana, Zimbabwe	QUANT Self-administered questionnaire, community-based	Mean age 26 years (range 18-65)<br><br>79% identified as black, 12% 'coloured', 6% white, 3% indian – representative of SA<br><br>52% without regular income	HIV prevalence 9.6% (self-reported)<br><br>47% had consensual sex with men	50.1% had used recreational drugs	31% of participants reported that they experienced forced sex at least once (14.9% reported forced sex by men only; 6.6% reported forced sex by women only; and 9.6% had forced sex experiences with both men and women)		Risk of bias unclear

Additional file 1

Author	Sample	Data collection method	Demographic information	Sexual health	Mental health	Violence	Other findings	
Smith, 2014 [39]	22 self-identified lesbian and bisexual women in South Africa (Cape Town)	QUAL Semi-structured interviews	Average age 23 years (range 19–32)<br><br>13 identified as black, 5 as 'coloured', 4 as white				All women stressed lack of sexual health information, and lack of health care providers' knowledge<br><br>'Most' women would not disclose their sexual orientation to health care provider	Quality high
Thurston et al. 2014 [36]	89 SM youth (61 women; overall sample of 818) in South Africa	QUANT Interviewer-administered questionnaire with stratified convenience sample	Mean age 17 years<br><br>58% identified as black, 11% as 'coloured', 5% as indian, 15% as white	40 SM youth were sexually active (not disaggregated by gender)	SM youth (69% SM women) showed higher levels of depression, traumatic stress and substance use than heterosexual matched peers.	SM youth (69% SM women) had experienced higher levels of partner-perpetrated violence than heterosexual matched peers.		Risk of bias unclear
Wells & Polders, 2006 [37]	487 self-identified gay, lesbian and bisexual people from Gauteng province, SA (45% SMW)	QUANT Self-administered questionnaire (closed-ended questions), stratified by race and gender	Mean age 27 years (range 16-59)<br><br>74% black SMW, 24% white SMW			36/40% (Black/White) SMW had experienced hate speech; 16/15% physical abuse/ assault; 10/4% sexual abuse/ rape; 17/8% domestic violence	Black SMW were more likely to experience violence in public spaces, and less likely to report homophobic violence to the police	Risk of bias unclear

QUAL = qualitative methodology; QUANT = quantitative methodology
