# Supplementary material for: Making the invisible visible: a systematic review of sexual minority women’s health in Southern Africa
Source: BMC Public Health. 2016 Apr 11;16:307. doi: 10.1186/s12889-016-2980-6 (PMC4827176; doi:10.1186/s12889-016-2980-6)
Supplement: Additional file 3: — Full search (PubMed). Details of full search strategy and results. (PDF 201 kb) [file 12889_2016_2980_MOESM3_ESM.pdf]

Additional File 2: Full search details

Search carried out in Pubmed for search range 01 January 2000 to 31 January 2015.

Search date 07 February 2015

#	*Searchers*	*Results*
1	"homosexuality, female"[MeSH Terms]	2,415
2	"homosexuality"[All Fields]	12,091
3	"female"[All Fields]	3,633,357
4	2 AND 3	5,574
5	"female homosexuality"[All Fields]	2,418
6	"lesbian"[All Fields]	2,866
7	1 OR 4 OR 5 OR 6	6,519
8	"bisexuality"[MeSH Terms]	2,069
9	"bisexuality"[All Fields]	2,148
10	"bisexual"[All Fields]	3,272
11	8 OR 9 OR 10	3,913
12	"women who have sex with women"[All Fields]	100
13	7 OR 11 OR 12	8,347
14	"hiv"[MeSH Terms]	51,818
15	"hiv"[All Fields]	187,668
16	14 OR 15	187,668
17	"depressive disorder"[MeSH Terms]	51,006
18	"depressive"[All Fields]	81,142
19	"disorder"[All Fields]	316,676
20	18 AND 19	58,022
21	"depressive disorder"[All Fields]	51,718
22	"depression"[All Fields]	168,089
23	"depression"[MeSH Terms]	53,381
24	17 OR 20 OR 21 OR 22 OR 23	184,317
25	"substance use"[All Fields]	17,108
26	"substance abuse"[All Fields]	30,419
27	"mental health"[All Fields]	108,847
28	"suicide"[MeSH Terms]	25,214
29	"suicide"[All Fields]	34,892
30	28 OR 29	35,576
31	"anxiety"[MeSH Terms]	35,209
32	"anxiety"[All Fields]	105,273
33	31 OR 32	105,637
34	"neoplasms"[MeSH Terms]	1,306,798
35	"neoplasms"[All Fields]	1,084,257
36	"cancer"[All Fields]	1,010,610
37	34 OR 35 OR 36	1,606,937
38	16 OR 24 OR 25 OR 26 OR 27 30 OR 33 OR 37	2,126,110
39	"South Africa"[All Fields]	43,479
40	"Southern Africa"[All Fields]	2,461
41	"africa"[MeSH Terms]	109,449
42	"africa"[All Fields]	84,629
43	41 OR 42	147,590
44	"namibia"[MeSH Terms]	430
45	"namibia"[All Fields]	810

46	44 OR 45	810
47	"zambia"[MeSH Terms]	1664
48	"zambia"[All Fields]	2594
49	47 OR 48	2594
50	"zimbabwe"[MeSH Terms]	1811
51	"zimbabwe"[All Fields]	2928
52	50 OR 51	2928
53	"lesotho"[MeSH Terms]	132
54	"lesotho"[All Fields]	298
55	53 OR 54	298
56	"botswana"[MeSH Terms]	815
57	"botswana"[All Fields]	1456
58	56 OR 57	1456
59	"swaziland"[MeSH Terms]	204
60	"swaziland"[All Fields]	386
61	59 OR 60	386
62	"mozambique"[MeSH Terms]	1006
63	"mozambique"[All Fields]	1812
64	62 OR 63	1812
65	39 OR 40 OR 43 OR 46 OR 49 OR 52 OR 55 OR 58 OR 61 OR 64	149,434
66	13 AND 38	4113
67	13 AND 38 AND 65	214
68	Limit 67 to English, French, Portuguese or German language	211
